# Broadly Defined Antibiotic Adjuvants for *Pseudomonas aeruginosa*

**DOI:** 10.1155/cjid/1388332

**Published:** 2026-08-21

**Authors:** Zhihan Chen, Yufan Li, Yuanlin Song

**Affiliations:** ^1^ Department of Pulmonary Medicine, Shanghai Key Laboratory of Lung Inflammation and Injury, Zhongshan Hospital, Shanghai Respiratory Research Institute, Fudan University, Shanghai, China, fudan.edu.cn; ^2^ Shanghai Institute of Infectious Disease and Biosecurity, Shanghai, China; ^3^ National and Shanghai Clinical Research Center for Aging and Medicine, Huashan Hospital, Fudan University, Shanghai, China, fudan.edu.cn; ^4^ Key Laboratory of Chemical Injury, Emergency and Critical Medicine of Shanghai Municipal Health Commission, Center of Emergency and Critical Medicine, Jinshan Hospital of Fudan University, Shanghai, China, jinshanhos.org.cn

**Keywords:** antibiotic adjuvants, antibiotics, efflux pump inhibitors, membrane permeabilizers, persister cells, *Pseudomonas aeruginosa*, quorum sensing inhibitors, siderophore analogs

## Abstract

*Pseudomonas aeruginosa* is a widely distributed opportunistic pathogen with multiple drug resistance, making it particularly challenging to treat in lower respiratory tract infections. Its resistance mechanisms are complex, including low outer‐membrane permeability, efflux pump overexpression, biofilm formation, and quorum sensing (QS) activation. Antibiotic adjuvants, as a strategy to enhance antibiotic efficacy and rapidly mitigate antibiotic resistance, have garnered significant attention. However, the traditional definition of antibiotic adjuvants typically refers to compounds like tazobactam, which lack inherent antibacterial activity but enhance the effects of antibiotics. This narrow definition emphasizes specific strategies to overcome *P. aeruginosa* resistance while excluding many promising approaches that enhance antibiotic activity through specific or nonspecific mechanisms. In this review, antibiotic adjuvants are defined as compounds coadministered or covalently linked with antibiotics to enhance the activity of the antibiotics. This review summarizes progress in research on “broadly defined antibiotic adjuvants” targeting *P. aeruginosa* and focuses on the following strategies: (1) enhancing outer‐membrane permeability to facilitate antibiotic entry into the bacterial cell; (2) efflux pump inhibitors (EPIs) to inhibit the bacterial efflux of antibiotics from the periplasm; (3) the “Trojan Horse” strategy, utilizing siderophores or their analogs to deliver antibiotics into bacteria; (4) quorum sensing inhibitors (QSIs), which weaken *P. aeruginosa* pathogenicity and resistance by blocking QS signaling pathways; and (5) activation of persister cells to enhance the bactericidal effects of antibiotics on persister cells in biofilm, facilitating the elimination of *P. aeruginosa* in chronic infections. These strategies provide a theoretical foundation for developing novel antibiotic adjuvants, which may contribute to addressing the challenge of *P. aeruginosa* resistance and improving clinical treatment outcomes.

## 1. Introduction

*Pseudomonas aeruginosa* is an opportunistic pathogen commonly encountered in clinical settings, characterized by widespread distribution, ease of colonization, strong resilience, and multiple drug resistance [1]. Risk factors for acute and chronic infections of the lower respiratory tract caused by *P. aeruginosa* include bronchiectasis, chronic obstructive pulmonary disease, cystic fibrosis, cancer, trauma, and immunocompromised states [2]. Direct triggers may include iatrogenic factors such as ventilator use, invasive procedures, implantation of artificial materials, and antibiotic use [3]. Beyond the respiratory tract, *P*. *aeruginosa* can also cause a broad spectrum of infections—including bloodstream, urinary tract, wound/burn, and device‐associated infections—particularly in patients with impaired host defenses, disrupted epithelial barriers, and exposure to invasive devices and prior broad‐spectrum antibiotics [4–6]. As an opportunistic pathogen, the activation of quorum sensing (QS) is a critical condition for *P. aeruginosa* infections. When bacterial density reaches a certain threshold, QS is activated, promoting the production of virulence factors and biofilm formation by *P. aeruginosa* [7]. Current treatment of *P. aeruginosa* infections relies primarily on antipseudomonal β‐lactams (with or without β‐lactamase inhibitors), fluoroquinolones, and aminoglycosides used depending on infection site, severity, and susceptibility results, whereas polymyxins (e.g., colistin) remain alternatives but are limited by toxicity concerns [8].

Despite the availability of various antibiotics, *P. aeruginosa* is notorious for its multidrug resistance (MDR). Due to its extensive intrinsic and adaptive resistance mechanisms, treating *P. aeruginosa* infections is highly challenging. In European populations, 12.9% of isolated *P. aeruginosa* strains exhibit combined resistance (resistant to three or more antimicrobial classes among piperacillin/tazobactam, ceftazidime, fluoroquinolones, aminoglycosides, and carbapenems) [9], whereas in China, the proportion of MDR *P. aeruginosa* among hospital‐acquired pneumonia (HAP) cases is as high as 27.9% [10]. In the United States, MDR *P. aeruginosa* was estimated to cause 32,600 cases and 2700 deaths among hospitalized patients in 2017, with an estimated $767 million in attributable healthcare costs [11]. The resistance mechanisms of *P. aeruginosa* are complex, with intrinsic mechanisms including low outer‐membrane permeability, overexpression of active efflux systems, and inactivating enzymes. Some adaptive resistance mechanisms require specific conditions, such as QS activation [12]. The unique resistance mechanisms and pathogenicity of *P. aeruginosa* also provide new avenues for treatment: targeting QS inhibition or activating persister cells to reduce pathogenicity and resistance, thereby enhancing antibiotic efficacy.

The concept of antibiotic adjuvants was initially established by β‐lactamase inhibitors, which restored β‐lactam activity and enabled multiple widely used combination therapies in clinical practice [8]. The traditional definition of antibiotic adjuvants typically refers to compounds like tazobactam, which lack inherent antibacterial activity but enhance the effects of antibiotics. More recently, the adjuvant paradigm has expanded beyond the traditional definition of antibiotic adjuvants. “Broadly defined antibiotic adjuvants” enhance antibiotic activity through specific or nonspecific mechanisms, regardless of whether they have intrinsic antibacterial activity. However, the development of “broadly defined antibiotic adjuvants” targeting *P. aeruginosa* still lacks systematic and purposeful approaches. This article focuses on *P. aeruginosa*–specific “broadly defined antibiotic adjuvants” and summarizes five types of drugs that can enhance antibiotic efficacy during *P. aeruginosa* treatment: (1) membrane permeabilizers; (2) efflux pump inhibitors (EPIs); (3) the “Trojan Horse” strategy; (4) quorum sensing inhibitors (QSIs); and (5) activation of persister cells, to clarify directions for future research and development. By cross‐comparing these approaches, we aim to highlight actionable design and combination opportunities and to clarify key translational hurdles (e.g., dose‐limiting toxicity, pharmacokinetics, and the need for well‐defined clinical indications) that must be addressed to accelerate the development and clinical deployment of adjuvants against MDR *P. aeruginosa*.

## 2. Main Drug Resistance Mechanisms of *P. aeruginosa*

### 2.1. Porins

Porins are the primary proteins associated with drug resistance on the outer membrane of *P. aeruginosa*. These porins typically exhibit low permeability and can be regulated in response to environmental changes [13]. They not only enhance *P. aeruginosa* resistance by reducing membrane permeability but also interact with the host environment, contributing to the formation of persister cells and biofilms [14, 15].

#### 2.1.1. OprF

OprF is a porin associated with nonspecific solute diffusion in the outer membrane of Gram‐negative bacteria, but the OprF of *P. aeruginosa* exhibits significantly lower permeability compared to other bacteria [16]. Structurally, OprF can adopt two stable conformers: an abundant two‐domain OmpA‐like conformer that is largely closed, and a rare one‐domain open‐channel conformer that folds into a larger β‐barrel and accounts for most channel activity. In reconstituted systems, only a small fraction (≈ 5%) of OprF molecules form open channels, providing a structural basis for the unusually low permeability of OprF in *P. aeruginosa* [17]. The low permeability of *P. aeruginosa*’s OprF contributes to resistance against small hydrophilic antibiotics, such as β‐lactams and tetracyclines, which cross the *P. aeruginosa* outer membrane predominantly via diffusion through water‐filled porin channels [16]. Subsequent studies have shown that OprF is highly expressed in hypoxic sputum environments and is essential for *P. aeruginosa* survival in the lungs of cystic fibrosis patients [18]. In hypoxic conditions, *P. aeruginosa* metabolism decreases, and the downregulation of the relatively high‐permeability OprD [15, 19], coupled with OprF upregulation, may help reduce outer‐membrane permeability, striking a balance between drug resistance and survival.

#### 2.1.2. OprD

OprD primarily contributes to *P. aeruginosa* survival by mediating the export of bacterial products and the uptake of nutrients via facilitated diffusion, while also allowing the entry of imipenem and meropenem [20, 21]. OprD on the outer membrane is regulated by outer‐membrane vesicles (OMVs) [22]. OprD can be carried by OMVs as they bud from the outer membrane, reducing outer‐membrane permeability, on one hand, and [23] enabling OMVs carrying OprD to absorb carbapenem antibiotics in the surrounding environment, on the other, thereby lowering antibiotic concentrations. Additionally, OprD can be sialylated by the host, reducing its binding affinity to β‐lactam antibiotics and decreasing permeability to piperacillin and ceftazidime [24].

#### 2.1.3. OprH

Lipopolysaccharides (LPS) carry negative charges and are stabilized by divalent‐cation bridges with Mg^2+^ and Ca^2+^. OprH can be overexpressed to replace Mg^2+^ and Ca^2+^ in forming stable cross‐links with LPS, reducing *P. aeruginosa* outer‐membrane permeability and effectively conferring resistance to positively charged antibiotics such as aminoglycosides and polymyxin B (PMB) [25]. However, this resistance mechanism may only play a supplementary role in environments deficient in Mg^2+^ [26].

### 2.2. Efflux Pumps

Metabolic by‐products or harmful compounds from the environment may enter the bacterial cell, necessitating effective efflux mechanisms to protect *P. aeruginosa*. *P. aeruginosa* possesses five main types of efflux pumps: (1) ATP‐binding cassette (ABC) superfamily, (2) major facilitator superfamily (MFS), (3) multidrug and toxic compound extrusion (MATE) family, (4) resistance‐nodulation‐division (RND) family, and (5) small multidrug resistance (SMR) family [2]. Among these, the RND family is most closely associated with drug resistance. Of the 12 RND efflux pumps, multidrug efflux AB‐outer‐membrane porin M (MexAB‐OprM), multidrug efflux CD‐outer‐membrane porin J (MexCD‐OprJ), multidrug efflux EF‐outer‐membrane porin N (MexEF‐OprN), and multidrug efflux XY‐outer‐membrane porin M (MexXY‐OprM) are of significant clinical relevance, contributing to *P. aeruginosa* drug resistance. The primary role of these efflux pumps in resistance is to expel antibiotics from the cytoplasm or periplasm to the extracellular environment [2].

#### 2.2.1. MexAB‐OprM

The MexAB‐OprM efflux pump expels a broad spectrum of antibiotics, including carbapenems, chloramphenicol, fluoroquinolones, lincomycin, macrolides, novobiocin, penicillins, tetracyclines, and all β‐lactams (excluding imipenem and biapenem), in addition to the antiseptic triclosan and the surfactant sodium dodecyl sulfate [27]. Recent mechanistic evidence shows that the endogenous phenazine pyocyanin can directly bind the MexAB‐OprM repressors MexR and NalD, promoting their dissociation from the mexA promoter and thereby derepressing efflux expression—linking virulence metabolite signaling (partially controlled by QS) to MDR regulation [28].

#### 2.2.2. MexCD‐OprJ

Studies have demonstrated that increased expression of the MexCD‐OprJ efflux pump strongly correlates with clinical *P. aeruginosa* resistance to ciprofloxacin, cefepime, and chloramphenicol [29].

#### 2.2.3. MexEF‐OprN

MexEF‐OprN can efflux chloramphenicol, fluoroquinolones, tetracycline, trimethoprim, and imipenem [30, 31]. Multidrug‐resistant nfxC‐type *P. aeruginosa* strains, which overexpress MexEF‐OprN, exhibit resistance to chloramphenicol, fluoroquinolones, tetracycline, trimethoprim, and imipenem [32].

#### 2.2.4. MexXY‐OprM

MexXY‐OprM is primarily responsible for the efflux of aminoglycosides, fluoroquinolones, and zwitterionic cephalosporins in *P. aeruginosa* [33]. High expression of MexXY‐OprM is frequently observed in cystic fibrosis patients [34, 35].

### 2.3. Bacterial Enzymes

Bacterial enzymes usually hydrolyze or modify antibiotics to protect *P. aeruginosa*. According to the antibiotics that enzymes target, bacterial enzymes can be divided into β‐lactamases and aminoglycoside‐modifying enzymes [36]. Some proteins can protect antibiotic targeting proteins from being affected, which is also included in bacterial enzymes. These include 16S rRNA methylases rendering the ribosome unaffected by aminoglycoside antibiotics [37], Qnr proteins protect DNA gyrase and topoisomerase IV from quinolone inhibition [38], and so on. Bacterial enzyme inhibitors are a kind of promising and generally applicable antibiotic adjuvant. Lots of its products have been used as first‐line medications [39]. Bacterial enzyme inhibitors represent a major and clinically advanced adjuvant modality. However, because they have been extensively covered in prior reviews and because this article emphasizes *P. aeruginosa*–specific permeability/efflux/uptake and antivirulence/tolerance strategies, we only briefly mention enzyme inhibition and refer readers to the literature for details [40, 41].

### 2.4. Adaptive Resistance

Adaptive resistance in *P. aeruginosa* refers to a temporary increase in the bacterium’s ability to withstand antimicrobial agents due to transient changes in gene and/or protein expression in response to environmental stimuli. This resistance is reversible upon removal of the triggering stimulus [1]. The most prominent mechanisms of adaptive resistance are biofilm formation and bacterial persistence [2]. These adaptive resistance mechanisms result in discrepancies between in vitro susceptibility testing and in vivo drug response. However, under specific inducing conditions, bacteria can alter their phenotype, offering opportunities for novel drug development strategies [1].

#### 2.4.1. Biofilm

Biofilm is defined as microbial communities that are irreversibly attached to a surface and enclosed in a matrix of primarily polysaccharide material [42]. Biofilm formation significantly enhances *P. aeruginosa* resistance to antibiotics, driven by mechanisms such as QS, reduced antibiotic penetration, altered metabolism, and the presence of persister cells [43]. Compared to planktonic *P. aeruginosa*, biofilm‐forming strains exhibit 100‐ to 1000‐fold greater tolerance to antimicrobial agents [44]. Biofilms are commonly observed in chronic cystic fibrosis lung infections and can also occur in patients with chronic obstructive pulmonary disease or bronchiectasis [45–48].

#### 2.4.2. Persister Cells

Persister cells are a subpopulation of slow‐growing, metabolically inactive bacteria within biofilms, typically comprising about 1% of the biofilm population, and exhibit high resistance to antibiotics [49]. These cells have reduced metabolic activity and downregulate the synthesis of many proteins, including antibiotic target sites [50]. Under antibiotic pressure, most *P. aeruginosa* cells are killed, but persister cells remain dormant. Once antibiotics are removed, persister cells resume growth, rapidly repopulating the biofilm. Consequently, persister cells are a major factor in the ability of chronic *P. aeruginosa* infections to resist antibiotic treatment and persist long term [50].

## 3. Antibiotic Adjuvants

### 3.1. Outer‐Membrane Permeabilizers

**FIGURE 1:**
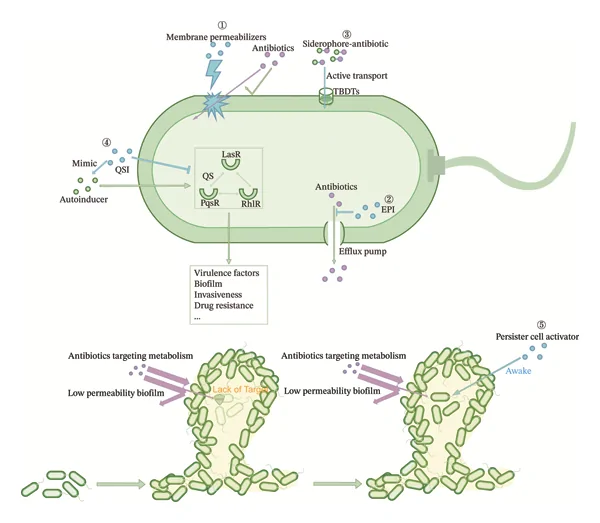
Antibiotic adjuvants using multiple strategies to enhance antibiotic effects. Green arrows denote the normal physiological processes of *P. aeruginosa*, purple arrows indicate the disruptive effects of antibiotics, and blue arrows represent the synergistic effects of antibiotic adjuvants. (1) Membrane permeabilizers damage *P. aeruginosa* membranes and facilitate the entry of antibiotics. (2) EPIs inhibit efflux pumps and elevate the concentration of antibiotics in *P. aeruginosa*’s cytoplasm. (3) Siderophore‐antibiotics are specifically identified and actively transported into *P. aeruginosa*’s periplasm. (4) QSIs mimic autoinducers and inhibit QS by competitive binding to QS‐related receptors, which regulate virulence factors, biofilm, invasiveness, drug resistance, and so on. (5) Influenced by factors such as hypoxia, nutrient deprivation, and quorum sensing, low‐metabolic persister cells emerge within the biofilm. Persister cells show strong resistance to antibiotics targeting metabolism. Persister cell activators stimulate these persister cells, thereby enhancing their susceptibility to antibiotics targeting metabolism. EPI: efflux pump inhibitor. TBDTs: TonB‐dependent transporters. QSI: quorum sensing inhibitor.

**FIGURE 2:**
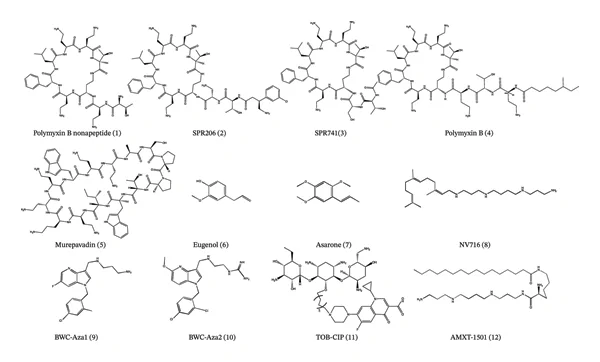
Membrane permeabilizers. 1–4: polymyxin B series membrane permeabilizers. 1–5: peptide‐based membrane permeabilizers. 6, 7: natural permeabilizers. 8–12: synthetic permeabilizers.

The low permeability of *P. aeruginosa*’s outer membrane poses a significant barrier to antibiotic efficacy, making the development of antibiotic adjuvants that enhance membrane permeability a promising strategy. However, the main problem of permeabilizers is specificity and cytotoxicity. Permeabilizers can be broadly categorized into peptides, metal‐based compounds, and small molecules. The general mechanism is shown in Figure 1, and all the permeabilizers are summarized in Table 1 and Figure 2.

**TABLE 1:** Outer‐membrane permeabilizers.

Compound	Specific target	Research status	Antibiotic adjuvant effects (reported^∗^)
Polymyxin B nonapeptide	LPS [51]	High toxicity, largely phased out [51]	Ciprofloxacin, norfloxacin, ofloxacin, and nalidixic acid [52]
SPR206	LPS [53]	Phase I [53]	NA [53]
SPR741	LPS [54, 55]	Phase I [54, 55]	Rifampin, erythromycin, and clarithromycin [54, 55]
Polymyxin B	LPS [56]	Phase IV [57]	Alkyl deoxyglycoside^∗∗^ [56]
P4‐9	NA [58]	Preclinical	Ceftazidime, fosfomycin, and erythromycin [58]
D‐11	LPS and membrane phospholipids [59]	Preclinical	Macrolides, rifampin family, colistin, tetracyclines, and quinolones [59]
Murepavadin	LptD [60]	Phase I [61, 62]	Ceftazidime/avibactam [60]
Pt NPs	OprD expression and other [63]	Preclinical	Imipenem and ciprofloxacin [63]
AGXX	ROS‐mediated damage [64]	Preclinical	Aminoglycosides and kanamycin [64]
AS101	ROS‐mediated damage [65]	Preclinical; Phase I/II trials for other indications [66, 67]	Mefloquine [65]
ZnO NPs	ROS‐mediated damage [68, 69]	Preclinical	Unclear [68, 69]
Eugenol	NA^∗∗∗^ [70]	Preclinical	Colistin [70]
Asarone	NA [71]	Preclinical; Phase II trials for Alzheimer’s disease [72]	Ampicillin [71]
NV716	NA [73]	Preclinical	Tetracycline [73]
BWC‐Aza1, BWC‐Aza2	NA [74]	Preclinical	Rifampin, erythromycin, and clarithromycin [74]
TOB–CIP	LPS and efflux pump [75]	Preclinical	Tetracycline [75]
*Artemisia argyi* essential oil	NA [76]	Preclinical	NA [76]
AMXT‐1501	NA [77]	Preclinical	NA [77]

*Note:* Synergy with other antibiotics may exist but has not been reported in the referenced literature. LPS: lipopolysaccharide. LptD: lipopolysaccharide transport protein D. Pt NPs: platinum nanoparticles. ZnO NPs: zinc oxide nanoparticles. TOB–CIP: tobramycin–ciprofloxacin.

Abbreviation: ROS, reactive oxygen species.

^∗^Data compiled from published studies.

^∗∗^Unable to penetrate Gram‐negative bacteria alone.

^∗∗∗^Have not been reported.

#### 3.1.1. Peptide‐Based Membrane Permeabilizers

Peptide‐based compounds are a significant and earliest focus for enhancing membrane permeability. Generally, peptide‐based membrane permeabilizers can be divided into PMB series, linear peptide chain, and other peptide‐based membrane permeabilizers. PMB series and linear peptide chains are cationic and amphipathic molecules, sharing similar mechanism. They are attracted to the anionic LPS layer of Gram‐negative outer membranes, competitively displace Mg^2+^/Ca^2+^ bridges that stabilize LPS, and create a “self‐promoted uptake” pathway that increases outer‐membrane permeability to otherwise excluded antibiotics [51, 78].

PMB series is the earliest kind of peptide‐based membrane permeabilizer. PMB compounds are a group of cyclic polypeptide antibiotics characterized by a conserved heptapeptide ring structure and a variable fatty acyl tail. The N‐terminal fatty acyl tail varies in length and composition, contributing to differences in membrane permeability effects and toxicity among the analog [79]. Back in 1971, Kubesch and colleagues demonstrated that PMB nonapeptide disrupts the outer membrane, significantly increasing *P. aeruginosa* sensitivity to ciprofloxacin, norfloxacin, ofloxacin, and nalidixic acid [52]. However, due to its high cytotoxicity, PMB nonapeptide is not considered for direct use in *P. aeruginosa* treatment [51]. SPR206, a PMB analog, acts independently against Gram‐negative bacteria [80] and has shown safety and tolerability in Phase I clinical trials, although its synergistic effects with other antibiotics remain unstudied [81]. Similarly, SPR741, another PMB nonapeptide analog, enhances outer‐membrane permeability in Gram‐negative bacteria, functioning either alone or in combination with antibiotics [53, 54, 82, 83]. PMB, used clinically as antibiotics, disrupts *P. aeruginosa* outer‐membrane integrity, facilitating the entry of alkyl deoxyglycoside to exert combined antibacterial effects [56]. Although PMB is called “last hope” antibiotic in some articles, *P. aeruginosa* may reduce its potentiating activity through ParR‐/ParS‐mediated LPS modifications, because PMB membrane permeabilizers rely on electrostatic interactions with LPS [78]. Such LPS modification potentially reduces surface negative charge, leading to cross‐tolerance with polymyxin‐like adjuvants.

As for linear peptide chain, a 13‐amino‐acid synthetic peptide, P4‐9 (PFWRIRIRRWWRR‐NH2), synergizes with ceftazidime and erythromycin against *P. aeruginosa*, with membrane damage requiring at least 2 h to repair, suggesting a prolonged synergistic effect akin to a post‐antibiotic effect [58]. Similarly, D‐11 (RLWKKIRQVIRK‐NH2), an all‐D‐amino‐acid peptide, enhances membrane permeability and inhibits efflux pumps, synergizing with antibiotics [59]. Other antimicrobial peptides, such as sp‐LECin (GCVFLLPAKPHNYKKVFLSKGV) [84] and Hill‐BB‐C10074 [85], increase *P. aeruginosa* membrane permeability, although their roles as antibiotic adjuvants remain unexplored.

Murepavadin, a peptide antibiotic, inhibits lipopolysaccharide transport protein D (LptD), compromising membrane integrity and synergizing with ceftazidime and avibactam [60]. With a specific target, Murepavadin showed better safety and entered Phase I clinical trial. Generally, membrane permeabilizers with specific targets such as LptD and LPS are more promising.

#### 3.1.2. Metal‐Containing Membrane Permeabilizers

Certain metal‐based compounds enhance antibiotic activity, which mostly damage membranes through reactive oxygen species (ROS). Studies have shown that prolonged exposure to platinum nanoparticles (Pt NPs) induces the overexpression of the OprD porin, preventing resistance to imipenem and ciprofloxacin [63]. AGXX, a metal nanoparticle‐based adjuvant, synergizes with aminoglycoside antibiotics and restores kanamycin sensitivity in kanamycin‐resistant *P. aeruginosa* strains [64]. AS101, a synthetic organotellurium compound containing tellurium (Te) with potent immunomodulatory and antimicrobial properties, stimulates ROS production to damage the cell wall [65] and has been evaluated in Phase I and II clinical trials for various conditions. Additionally, zinc oxide nanoparticles (ZnO NPs) have been shown to increase both inner and outer‐membrane permeability in *P. aeruginosa* [68, 69], although its potential as antibiotic adjuvants requires further investigation. One of the advantages of ROS‐generating metal‐based permeabilizers is that stable high‐level resistance appears less straightforward, yet adaptive tolerance mediated by oxidative‐stress responses, QS, and biofilm formation may reduce efficacy, particularly under low‐dose exposure [86–88].

#### 3.1.3. Natural or Synthetic Small‐Molecule Membrane Permeabilizers

Numerous synthetic and naturally derived small molecules enhance *P. aeruginosa* membrane permeability, although most of them lack specificity. Examples include eugenol [70], asarone (*Acorus calamus* rhizome extract) [71], Compound NV716 (a polyaminoisoprenyl molecule) [73], and BWC‐Aza1/2 (azaindole compounds) [74]. Tobramycin–ciprofloxacin (TOB–CIP) is a new concept compound, where TOB part can specifically interact with LPS, while CIP can inhibit efflux pump and thus synergize with tetracycline and quinolones [75]. Additionally, many compounds such as *Artemisia argyi* essential oil [76] and AMXT‐1501 [77] have been shown to increase *P. aeruginosa* membrane permeability, but their antibiotic adjuvant effects require further study. Finally, antibacterial blue light disrupts *P. aeruginosa* membranes, aiding antibiotics like chloramphenicol and linezolid, but its use is currently limited to topical wound infections [89].

### 3.2. EPIs

**FIGURE 3:**
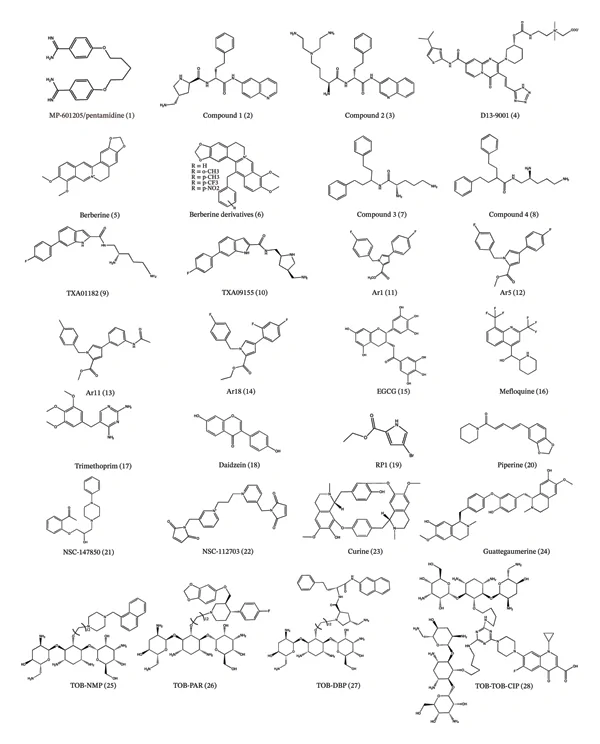
Efflux pump inhibitors. 1: MP‐601205/pentamidine. 2, 3: PAβN series. 4: pyridopyrimidinone series. 5, 6: berberine and its derivatives. 7–10: aryl indole carboxamide compounds. 11–14: pyrrole core–based EPIs. 15–24: EPIs identified by screening of natural and synthetic molecular databases. 25–28: TOB covalent conjugate EPIs.

Efflux pumps contribute to *P. aeruginosa* resistance against various antibiotics. EPIs increase antibiotic concentrations in the periplasm of *P. aeruginosa*, thereby enhancing antibiotic efficacy. Currently, no EPIs have been approved for clinical use, except for MP‐601205 (also called pentamidine), which was studied in clinical trials but was discontinued due to tolerability issues. Nevertheless, several promising EPIs are still under investigation. The general mechanism is shown in Figure 1, and all the EPIs are summarized in Table 2 and Figure 3.

**TABLE 2:** Efflux pump inhibitors.

Compound	Mechanism of action	Research status	Antibiotic adjuvant effects (reported^∗^)
MP‐601205 (pentamidine)	Inhibits RND efflux pumps; membrane permeabilization [90]	Phase Ib [91]	Levofloxacin [91], ciprofloxacin [90]
PAβN	Inhibits multiple RND efflux pumps [92]	Preclinical	Levofloxacin, aztreonam, and ciprofloxacin [92]
Compound 1	Inhibits multiple RND efflux pumps [93]	Preclinical	Levofloxacin [93]
Compound 2	Inhibits multiple RND efflux pumps [94]	Preclinical	Levofloxacin [94]
D13‐9001	Inhibits MexAB‐OprM [95, 96]	Preclinical	Levofloxacin and aztreonam [97]
Reserpine	Psychotropic drug; inhibits RND efflux pumps [98]	Preclinical; clinical drug for other indications	Levofloxacin [98]
Verapamil	Calcium channel blocker; inhibits RND and ABC efflux pumps [98]	Preclinical; clinical drug for other indications	Carbenicillin, neomycin, and erythromycin [98]
Sertraline	Psychotropic drug; inhibits RND efflux pumps [99]	Preclinical; clinical drug for other indications	Levofloxacin [99]
Digitoxin	Ion channel inhibitor; inhibits RND efflux pumps [100]	Preclinical; clinical drug for other indications	Levofloxacin [100]
Fluoxetine	Psychotropic drug; inhibits RND efflux pumps [101]	Preclinical; clinical drug for other indications	Ciprofloxacin, gentamicin, meropenem [101]
Promethazine	Psychotropic drug; inhibits RND efflux pumps [101]	Preclinical; clinical drug for other indications	Ciprofloxacin, gentamicin, meropenem [101]
Chlorpromazine	Psychotropic drug; inhibits RND efflux pumps [102]	Preclinical; clinical drug for other indications	Chloramphenicol, ciprofloxacin, tetracycline, and nalidixic acid [102]
Amitriptyline	Psychotropic drug; inhibits RND efflux pumps [102]	Preclinical; clinical drug for other indications	Chloramphenicol, nalidixic acid, and tetracycline [102]
Berberine	Inhibits MexXY‐OprM [103]	Preclinical	Tobramycin [103]
Berberine derivatives	Inhibits MexXY‐OprM [104]	Preclinical	Tobramycin [104]
TXA01182	Inhibits RND efflux pumps; mild membrane permeabilization [105]	Preclinical	Levofloxacin, sulbactam, fluoroquinolones, sulfonamides, and tetracyclines [105]
TXA09155	Conformationally constrained aryl indole carboxamide; similar to PAβN [106]	Preclinical	Levofloxacin, sulbactam, fluoroquinolones, sulfonamides, and tetracyclines [106]
Ar1, Ar5, Ar11, Ar18	Pyrrole‐core EPIs; bind AcrB and MexB [107]	Preclinical	Tetracycline and levofloxacin [107]
EGCG	Competes with MexAB‐OprM; may have intrinsic antibacterial activity [108, 109]	Preclinical	Aztreonam, ciprofloxacin [108, 109]
Mefloquine	Inhibits RND efflux pumps [110]	Preclinical; clinical drug for other indications	NA^∗∗^ [110, 111]
Trimethoprim	Inhibits RND efflux pumps [112]	Preclinical; clinical drug for other indications	Levofloxacin [112, 113]
Daidzein	Plant‐derived; inhibits RND efflux pumps [100]	Preclinical	Levofloxacin [100]
RP1	Binds MexAB‐OprM with high affinity [114]	Preclinical	Levofloxacin [114]
Piperine	Low‐affinity binding to MexAB‐OprM; suppresses expression [115]	Preclinical	Imipenem [115]
NSC‐147850, NSC‐112703	inhibit RND pumps [116]	Preclinical	NA [116]
Curine	Inhibit ABC transporter [117]	Preclinical	Carbenicillin, neomycin, and erythromycin [117]
Guattegaumerine	Inhibit ABC transporter [117]	Preclinical	Carbenicillin, neomycin, and erythromycin [117]
TOB–NMP, TOB–PAR, TOB–DBP	Inhibit RND efflux pumps [118]	Preclinical	Neomycin, rifampicin, moxifloxacin, ciprofloxacin, and fosfomycin [118]
TOB–TOB–CIP	Inhibit efflux pumps [119]	Preclinical	Ceftazidime, aztreonam, imipenem, meropenem, ceftolozane [119]

*Note:* Synergy with other antibiotics may exist but has not been reported in the referenced literature. EGCG: epigallocatechin gallate. RP1: 4‐bromopyrrole‐2‐carboxylate. TOB: tobramycin. NMP: 1‐(1‐naphthylmethyl)‐piperazine. PAR: paroxetine. DBP: dibasic peptides. CIP: ciprofloxacin.

^∗^Data compiled from published studies.

^∗∗^Have not been reported.

#### 3.2.1. PAβN Series

The PAβN series represents the most extensively studied class of EPIs, having undergone systematic structure–activity relationship (SAR) studies [120, 121]. Lamut et al. summarized this process in detail [92]. PAβN series exhibit broad‐spectrum and potent efflux pump inhibition, effective against both *P. aeruginosa* and *Escherichia coli*. But even after extensive SAR studies, their high toxicity remains a challenge. Up to now, two of the most promising compounds are compound 1 [93] and Compound 2 [94]. Compound 2 was identified through extensive molecular screening, coincidentally sharing a chemical structure similar to PAβN series [94]. In mice, intravenous administration of PAβN and related analogs yields an LD_50_ below 25 mg/kg; more problematically, the “basic side chain” is both essential for efflux‐inhibitory activity and a principal driver of toxicity [95]. The series’ advancement was largely suspended by the mid‐2000s due to persistent toxicological profiles and no compounds progressed to clinical trial [95].

#### 3.2.2. Pyridopyrimidinone Series

The pyridopyrimidinone series were developed mostly by Nakayama and his colleagues. The pyridopyrimidinone series specifically inhibits the MexAB‐OprM efflux pump, limiting its effectiveness against *P. aeruginosa* strains overexpressing other RND efflux pumps. A thiazole moiety and an acidic group are essential structural components of pyridopyrimidinones [92]. After addressing its high binding to serum proteins [122, 123], adding groups to fit the hydrophobic pocket region, improving water solubility and periplasmic entry [124–127], D13‐9001 was synthesized. D13‐9001, creatively incorporating a quaternary ammonium moiety, achieved high water solubility, strong binding affinity, low toxicity, and favorable pharmacokinetics. In the original lead‐optimization study, D13‐9001 was not lethal to mice at 100 mg/kg in an acute toxicity assay and was therefore selected for further in vivo profiling [97]. Despite these advancements, the high specificity of pyridopyrimidinones for MexAB‐OprM limits their broader application, and issues with comprehensive safety liabilities relevant to clinical translation continue to hinder clinical development [95, 96]. Recent studies continue to explore PAβN and D13‐9001 as standard EPIs, focusing on low‐cost production and recalibration of synergistic effects to support further EPI research [128–130].

As classical EPIs, D13‐9001 and PAβN have been used as representative probes of MexAB‐OprM–specific versus broad‐spectrum EPI classes, respectively, to study the evolution of EPI resistance in *P. aeruginosa*. Resistance to D13‐9001 in *P. aeruginosa* appears to arise mainly via (1) on‐target mutations (e.g., MexB F628L, which abolishes inhibition by D13‐9001) [96] and (2) bypass via alternative efflux, for example, PA1438 (L172P)‐associated > 150‐fold upregulation of MexMN that enables MexMN‐OprM to substitute for MexAB‐OprM and thereby diminishes D13‐9001 adjuvant activity [96]. For broad‐spectrum EPI, PAβN, direct resistance‐evolution evidence is less developed, but its competitive and membrane‐permeabilizing features suggest that common efflux‐regulatory mutations and permeability remodeling in *P. aeruginosa* could attenuate adjuvant performance [131, 132].

#### 3.2.3. Ion Channel and Psychotropic Drugs

Ion channel and psychotropic drugs are often found to be EPIs. It is assumed that ion channels share a similar structure with efflux pumps [98, 100, 117] and psychotropic drugs frequently exhibit amphiphilic structures, which is essential for crossing the blood–brain barrier, capable of binding RND efflux pumps [99, 101, 102]. In thiophene and tricyclic drugs, the hydrophobic core is particularly suited for binding RND hydrophobic pockets [101, 102]. Reserpine and verapamil were among the earliest identified EPIs [98]. After 2011, the psychotropic drug sertraline was shown to have EPI activity in *E. coli* and *P. aeruginosa* [99, 113]. Digitoxin [100], fluoxetine, promethazine [101], chlorpromazine, and amitriptyline [102] were also found to exhibit EPI activity. However, their EPI activity generally requires concentrations far above safe in vivo levels, limiting their role to guiding EPI design [101, 102].

#### 3.2.4. Berberine and Its Derivatives

In 2019, Galeazzi’s team identified the EPI activity of berberine and conducted structural modifications. Berberine was demonstrated high affinity for MexY and synergized with TOB to inhibit *P. aeruginosa* at 80 μg/mL [103]. However, this concentration is toxic in vivo, and oral administration of berberine cannot achieve the required plasma levels [104]. Subsequent modifications by Galeazzi’s team showed that introducing lipophilic groups at the 13‐C position of berberine enhances the EPI activity of its derivatives, although their effective concentrations remain high [133, 134].

#### 3.2.5. Aryl Indole Carboxamide Compounds

LaVoie et al. synthesized Compounds 3 and 4, which are two‐aryl alkyl diaminopentanamides, demonstrating synergy with clarithromycin in *E. coli*. These compounds bind RND pumps but also exhibit mild membrane permeabilization, contributing to excessive membrane toxicity [135, 136]. To address this, the team replaced the two‐aryl alkyl group with an aryl indole, synthesizing TXA01182. At 6.25 μg/mL, TXA01182 reduced the minimum inhibitory concentration (MIC) of levofloxacin against *P. aeruginosa* by eightfold and showed synergy with various clinical isolates, as well as with sulbactam, fluoroquinolones, sulfonamides, and tetracyclines. TXA01182’s membrane toxicity was lower than that of amphotericin B [105]. Further improvements using a conformationally constrained approach led to TXA09155, which features an aryl indole head and a tail similar to PAβN compounds, with slightly superior synergistic effects compared to TXA01182 [106].

#### 3.2.6. Pyrrole Core–Based EPIs

In 2024, Nandanwar and colleagues reported a class of EPIs with a pyrrole ring core, characterized by a pyrrole core, two hydrophobic aromatic ring tails, and a polar head. Compounds Ar1, Ar5, Ar11, and Ar18, at 16 and 8 μg/mL, reduced the MIC of tetracycline against *P. aeruginosa* from 64 to 2 μg/mL [107]. These pyrrole‐core EPIs specifically bind AcrB and MexB, demonstrating good tolerability and synergy in mammalian tolerability tests and mouse lung infection models [107]. Structurally distinct from pyridopyrimidinones, these promising compounds await further development.

#### 3.2.7. Screening of Natural and Synthetic Molecular Databases

As early as 2004, epigallocatechin gallate (EGCG) was found to exhibit EPI activity in *Staphylococcus aureus*, synergizing with tetracycline [108]. Further studies revealed that at high concentrations, EGCG directly inhibits *P. aeruginosa*, while at subinhibitory concentrations, it synergizes with PAβN, aztreonam, and ciprofloxacin. This synergy is more pronounced in MexAB‐OprM–deficient *P. aeruginosa*, suggesting that EGCG may compete with MexAB‐OprM for efflux, while also possessing intrinsic antibacterial activity [109]. In 2009, Vidal‐Aroca and colleagues developed a resazurin‐based method to screen EPIs [110], identifying mefloquine as an EPI, though its effective concentration exceeded safe levels [111]. In 2010, the FDA‐approved drug trimethoprim was shown to have EPI properties [112, 113]. In 2014, daidzein, a plant‐derived compound involved in secondary metabolism and defense responses, was identified as EPI in *P. aeruginosa* [100]. While daidzein’s EPI activity extended beyond *P. aeruginosa*, its role in inhibiting efflux pumps in mycobacteria and cancer cells garnered more attention [137]. With advancements in computational technology, large‐scale molecular simulations have become an efficient screening method. In 2022, Nandanwar and colleagues used computational screening of over 4000 microbial products to identify 4‐bromopyrrole‐2‐carboxylate (RP1), which binds MexAB‐OprM with high affinity. RP1 exhibited strong antibiotic synergy and tolerability, improving levofloxacin’s efficacy in a mouse model of *P. aeruginosa* lung infection [114]. In 2023, piperine’s EPI activity in *P. aeruginosa* was studied, showing low‐affinity binding to MexAB‐OprM and suppression of its expression at the transcriptional level. At high concentrations, piperine synergized with imipenem, but its safety and specificity warrant further investigation [115]. In 2024, another computational study identified NSC‐147850 and NSC‐112703, which differ structurally from pyridopyrimidinones but exhibit EPI activity and antibiotic synergy, although their toxicity requires further validation [116].

#### 3.2.8. ABC Transporter Inhibitory Molecules

Although RND efflux pumps are the primary efflux mechanism in *P. aeruginosa*, resistance to antibiotics such as carbenicillin, neomycin, and erythromycin is partly mediated by ABC efflux pumps [117]. Few studies have focused on ABC transporter inhibitors in *P. aeruginosa*. A 2022 study reported that verapamil, curine, and guattegaumerine, known to inhibit eukaryotic ABC transporters, also inhibit *P. aeruginosa*’s ABC transporters. In neomycin‐resistant *E. coli*, 30% of resistance is attributed to the MsbA pump, and these compounds specifically bind MsbA, enhancing neomycin’s activity [117]. They also increase *P. aeruginosa*’s sensitivity to carbenicillin, neomycin, and erythromycin, although their effects on RND pumps have not been ruled out [117].

#### 3.2.9. Enhancing EPIs Through Covalent Conjugation or Coadministration

Besides the inhibitory activity of EPIs on efflux pumps, EPI efficacy is also affected by whether the compound can enter and accumulate inside *P. aeruginosa* to reach the pump’s internal binding site. Enhancing EPI penetration through the *P. aeruginosa* outer membrane and their ability to interfere with efflux pumps can improve synergy with antibiotics [118, 119, 138]. 1‐(1‐Naphthylmethyl)‐piperazine (NMP), paroxetine (PAR), and dibasic peptides (DBPs) are known EPIs with limited activity; NMP and PAR are ineffective against *P. aeruginosa*. TOB, an aminoglycoside, has a unique mechanism for entering Gram‐negative bacteria. TOB is strongly cationic and can bind anionic sites on LPS, competitively displacing divalent cations and transiently disrupting the *P. aeruginosa* outer membrane to access the periplasm. Research also shows conjugating TOB with EPIs may enhance the inhibitory activity of EPIs [138]. Yang and colleagues synthesized TOB–NMP, TOB–PAR, and TOB–DBP, all of which synergized with minocycline against *P. aeruginosa*, with DBP showing enhanced synergy. These conjugates also synergized with neomycin, rifampicin, and other antibiotics, though toxicity data were not provided [118]. In 2023, an innovative idea synthesized TOB–TOB–CIP (ciprofloxacin). Although CIP lost its antibacterial activity, TOB–TOB–CIP readily enters *P. aeruginosa* and competes with other antibiotics for efflux pumps. Toxicity reports indicated high cell viability (> 90% in HEK293 cells and > 80% in Hep G2 cells) at effective concentrations [119]. Additionally, a recent study showed that liposomal delivery of antibiotics combined with EPIs reduced the MIC of free gentamicin against *P. aeruginosa* from 256 to 8 μg/mL, within the CLSI intermediate range for gentamicin versus *P. aeruginosa* [139]. Combining multiple strategies may lower antibiotic MICs to susceptible ranges without significantly increasing toxicity.

#### 3.2.10. Antibacterial Substances Requiring EPIs to Exert Effects

EPI research is not limited to synergizing with existing antibiotics. Some antibacterial substances, being ineffective due to efficient efflux, can achieve unexpected effects when combined with EPIs. A 2024 study screened antibacterial substances in the presence of PAβN as an EPI and found that 5‐O‐mycaminosyltylonolide, ineffective alone against *P. aeruginosa*, exhibited a strong antibacterial activity when combined with PAβN. This is likely due to its susceptibility to efficient *P. aeruginosa* efflux [140]. However, this activity requires a high PAβN concentration (below its own antibacterial threshold) [140], and the lack of clinically effective EPIs limits the practical application of such studies, which depend on further EPI development.

#### 3.2.11. Challenges of MP‐601205 and Repurposed Drugs as EPIs

Pentamidine is an approved antiparasitic agent (approved for intravenous injection, intramuscular injection, and inhalation administration), and it was identified as having EPI activity [141]. For commercial confidentiality reasons, MPEX Pharmaceuticals initially referred to pentamidine as MP‐601205 and conducted a series of studies, which also contributed to inconsistent naming in the EPIs literature [142]. Before 2020, published information about MP‐601205 was limited, mostly came from a patent (US7947741B2) disclosed in 2011. The patent revealed that MP‐601205 was considered an EPI candidate (in vitro potentiation was observed at ∼10–20 μg/mL) and was evaluated as an inhaled adjunct for *P. aeruginosa* infections in patients with cystic fibrosis in combination with levofloxacin, but the Phase 1b program was terminated early due to tolerability issues [141, 143]. In recent years, pentamidine has regained attention and its systematic basic papers appear, pointing out pentamidine potentiates ciprofloxacin by enhancing membrane permeability and inhibiting efflux pump [90, 144].

Several compounds reported to exhibit EPI activity are approved drugs for other indications. However, because they were not optimized through dedicated SAR campaigns to increase affinity for bacterial efflux pumps, their EPI effects typically require concentrations far exceeding those used for their original clinical indications. Consequently, such repurposed agents often serve mainly as starting points or structural inspiration for subsequent EPI design rather than as directly translatable EPI candidates.

#### 3.2.12. Translational Barriers and Future Prospects for EPIs

Despite the continued emergence of new EPIs, clinical translation remains limited because the key hurdles—potency at clinically achievable exposures, target specificity, and systemic tolerability—are tightly coupled rather than independently optimizable [145]. At present, broad‐spectrum EPIs often face the challenge of potency at clinically achievable exposures, whereas MexB‐specific EPIs must contend with concerns regarding their inability to inhibit MexMN‐OprM and the risk of resistance development [96]. The combined use of membrane permeabilizers and EPIs may represent a practical approach to address the issue of potency at clinically achievable exposures. Mechanistically, their synergy can be understood at two levels. First, membrane permeabilizers facilitate EPI entry, thereby enhancing the apparent potency of EPIs. Second, the simultaneous disruption of the membrane barrier function and suppression of active efflux can accelerate antibiotic accumulation in *P*. *aeruginosa*. In this way, such combinations can produce synergy while allowing each component to be used at lower concentrations. For example, Ferrer‐Espada et al. reported that in some strains, 1 μg/mL PMB nonapeptide plus 1 μg/mL PAβN reduced the MIC of azithromycin from 128 to 0.06 μg/mL [101], whereas PAβN typically requires ∼20–50 μg/mL to potentiate antibiotics when used alone [95]. Thus, membrane permeabilizer–EPI combination regimens merit further exploration. In parallel, refined SAR to reduce the concentrations required for potentiation remains a central direction. Given the presence of nucleophilic residues Tyr/Lys/Thr/Ser and the basic microenvironment shaped by Arg in the hydrophilic region of the MexB pocket [146, 147], incorporating a SuFEx warhead to enable covalent engagement may be a worthwhile design hypothesis to test.

### 3.3. Trojan Horse Strategy

Siderophore‐dependent iron‐uptake systems represent a critical pathway for *P. aeruginosa* to acquire iron, while also serving as a potential vulnerability in its highly impermeable outer membrane, often described as its “Achilles’ heel.” Conjugating antibiotics to siderophores, through either stable or degradable linkages, constitutes an effective “Trojan Horse” strategy to enhance antibiotic delivery [148]. Notably, naturally occurring sideromycins, which integrate siderophore and antibiotic domains, exemplify this “Trojan Horse” strategy in nature [149].

*P. aeruginosa* primarily secretes two siderophores: pyoverdine (PVD) and pyochelin (PCH). PCH is typically utilized for iron uptake under general iron‐limiting conditions, while PVD is employed under severe iron restriction [150]. Additionally, *P. aeruginosa* can exploit catechol‐type siderophores produced by other bacteria, actively transporting them into the periplasm via TonB‐dependent receptors (TBDRs) such as FpvA, FptA, PfeA, PirA, and FvbB [151, 152]. In 2014, Mislin and colleagues reviewed *P. aeruginosa*’s siderophore systems and existing siderophore‐antibiotic conjugates, outlining key features for designing effective conjugates [148]. They noted that *P. aeruginosa* produces three distinct PVD variants and selectively transports its corresponding PVD, rendering PVD‐antibiotic conjugates less feasible [148]. Similarly, PCH‐antibiotic conjugates have shown limited success; for instance, Rivault’s team synthesized two PCH‐norfloxacin conjugates with hydrolyzable linkers, which exhibited antibacterial activity against *P. aeruginosa* but with MICs higher than free norfloxacin [153]. Likewise, Yoganathan’s team synthesized a PCH‐C5‐linked gallidermin conjugate, showing no activity against *P. aeruginosa* [154].

**FIGURE 4:**
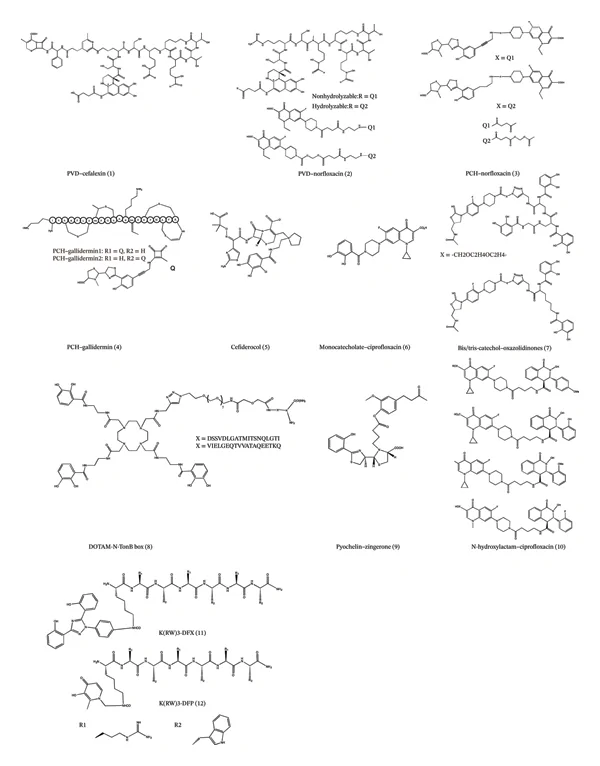
Siderophore‐antibiotic strategy. 1, 2: PVD‐based siderophore‐antibiotic conjugates. 3, 4, 9: PCH‐based siderophore‐antibiotic conjugates. 5–8: catechol‐based siderophore‐antibiotic conjugates. 10: N‐hydroxylactam‐based siderophore‐antibiotic conjugates. 11, 12: nonsiderophore iron chelators.

However, a breakthrough occurred in 2015 with the development of cefiderocol [155], a semisynthetic antibiotic featuring a catechol moiety that binds iron and is actively taken up by *P. aeruginosa*. Structurally similar to ceftazidime and cefepime, cefiderocol is clinically used to treat challenging *P. aeruginosa* infections [156], demonstrating efficacy against 99% of carbapenem‐nonsusceptible *P. aeruginosa* strains [8]. Although many other attempts have yielded suboptimal results, these efforts have significantly advanced our understanding of the “Trojan Horse” strategy. The general mechanism is shown in Figure 1, and all the siderophore‐antibiotics are summarized in Table 3 and Figure 4.

**TABLE 3:** Siderophore‐antibiotic.

Compound	Type	Stage	Effect
PVD–cefalexin	Pyoverdine‐based [157]	Preclinical	Induced bacterial growth [157]
PVD–norfloxacin	Pyoverdine‐based [158]	Preclinical	MIC higher than free norfloxacin, regardless of hydrolyzable or nonhydrolyzable linker, as norfloxacin requires cytoplasmic entry for activity [158]
PCH–norfloxacin	Pyochelin‐based [153]	Preclinical	MIC higher than free norfloxacin, regardless of hydrolyzable or nonhydrolyzable linker [153]
PCH–gallidermin	Pyochelin‐based [154]	Preclinical	Slightly promoted *P. aeruginosa* growth [154]
Cefiderocol	Catechol‐based [156]	Phase III Clinical	Equal or superior to ceftazidime‐avibactam and meropenem Siderophore‐like moiety and resistance to β‐lactamase inactivation enable treatment of MDR *P. aeruginosa* [156]
Monocatecholate–ciprofloxacin	Catechol‐based [159]	Preclinical	Equivalent to free ciprofloxacin against AM85 in SMM with 1 μM FeCl_3_; ineffective in the M–H medium [159]
Bis/Tris–catechol–oxazolidinones	Catechol‐based [160]	Preclinical	Ineffective against *P. aeruginosa* [160]
DOTAM‐N‐TonB box	Catechol‐based [161]	Preclinical	Effective against ΔpvdFΔpchA mutants but ineffective against PAO1 [161]
Pyochelin–zingerone	Pyochelin‐based [162]	Preclinical	More effective than zingerone alone [162]
N‐Hydroxylactam–ciprofloxacin	N‐Hydroxylactam‐based [163]	Preclinical	Effective only against ΔpvdFΔpchA mutants [163]
K (RW)_3_‐DFX, K (RW)_3_‐DFP	Nonsiderophore iron chelators [164]	Preclinical	MIC of 2 μM, significantly lower than 64 μM for free K (RW)_3_ [164]

*Note:* PVD: pyoverdine. PCH: pyochelin. DFX: deferasirox. DFP: deferiprone–carboxylate.

#### 3.3.1. Siderophore‐Antibiotic Strategy

The siderophore‐antibiotic strategy is the most traditional “Trojan Horse strategy.” Conjugating siderophore analogs with antibiotics is theoretically a good approach to overcome the low outer‐membrane permeability of *P. aeruginosa*, particularly because siderophore can carry large molecules like peptides. Accordingly, some peptide inhibitors were also tried to link with siderophores.

Catechol series mimics enterobactin, which is produced by *E. coli* and can be used by *P. aeruginosa*. Cefiderocol is a notable success. Similarly, Fardeau and colleagues synthesized several tricatecholate–ciprofloxacin conjugates mimicking enterobactin, which exhibited vectorization (enhanced activity in the presence of FeCl_3_ in the succinate minimum medium, SMM), but their activity against *P. aeruginosa* was lower than free ciprofloxacin [159]. Interestingly, a nonhydrolyzable monocatecholate–ciprofloxacin conjugate showed vectorization and an MIC equivalent to free ciprofloxacin against *P. aeruginosa*, suggesting that monocatecholate may enter the cytoplasm, unlike enterobactin, which remains in the periplasm [159].

This strategy also enables antibiotics typically used against Gram‐positive bacteria or large molecules to be considered for *P. aeruginosa* treatment. Oxazolidinones, primarily effective against Gram‐positive bacteria due to poor Gram‐negative penetration, were conjugated with bis/tris‐catechol by Moynié and colleagues, but the conjugates lacked activity against *P. aeruginosa* [160]. This may be due to the low affinity of bis/tris‐catechol for the PfeA receptor or steric hindrance of the oxazolidinone by the siderophore moiety [160]. In another research, protein‐based inhibitors, TonB box, was conjugated with MECAM and DOTAM. However, only some DOTAM‐N‐TonB box conjugates were effective against ΔpvdFΔpchA mutants, not PAO1 [161], indicating that PAO1 preferentially uptakes its own siderophores [165, 166]. The TonB box design highlights the potential of the “Trojan Horse” strategy to make periplasmic proteins accessible targets for protein‐based inhibitors, expanding therapeutic options against *P. aeruginosa*. Siderophores have also been conjugated with bacteriostatic natural extracts to enhance their activity. For example, PCH–zingerone conjugates exhibited stronger QS and biofilm inhibition than zingerone alone [162].

N‐Hydroxylactam is another siderophore core utilized by *P. aeruginosa*. Bakulina and colleagues synthesized four N‐hydroxylactam‐based siderophore analogs, one of which (DVD‐000304) matched PVD’s transport efficiency [163]. However, its ciprofloxacin conjugate was ineffective against PAO1, showing activity only against ΔpvdFΔpchA mutants, indicating the need for siderophores to compete with PVD for receptor binding.

Nonsiderophore iron chelators may also serve as “Trojan Horse” vectors, potentially via nonsiderophore‐dependent pathways. Olshvang and colleagues conjugated K (RW)_3_, a peptide typically active against Gram‐positive bacteria, with nonsiderophore chelators deferasirox (DFX) and deferiprone–carboxylate (DFP). In the casamino acid medium (CAA), K (RW)_3_‐DFX and K (RW)_3_‐DFP achieved MICs of 2 μM, significantly lower than the 64 μM of free K (RW)_3_. However, ^55^Fe experiments indicated that DFX does not transport iron into *P. aeruginosa*, suggesting an unknown mechanism for its entry [164].

#### 3.3.2. Fundamental Research on Siderophores

Fundamental research on siderophores provides critical guidance for developing siderophore–antibiotic and xenometallomycin drugs. Current studies focus on developing novel siderophore analogs, characterizing *P. aeruginosa* siderophore receptor proteins, and elucidating siderophore‐*P. aeruginosa* interactions.

For example, although PVD cannot be artificially synthesized, Antonietti and colleagues synthesized PVD analogs aPvd2 and aPvd3, both capable of transporting iron into *P. aeruginosa*. However, aPvd2 only promoted PAD07 growth, while aPvd3 supported PAO1 growth. Many siderophore analogs struggle to compete with PVD for receptor binding [167]. Studies on ferrioxamine B and ferrichrome, which enter *P. aeruginosa* via the FpvB receptor, revealed that they share a binding site with PVD but have distinct binding mechanisms and higher affinity for FpvB. While both promote *P. aeruginosa* growth, their efficacy is influenced by PVD concentration [168]. Additionally, Zhang and colleagues explored the de novo synthesis of pseudopaline, a metallophore favoring zinc, nickel, and cobalt uptake, which may also serve as a “Trojan Horse” candidate [169].

Regarding *P. aeruginosa* receptors, PiuD, a homolog of PiuA, was identified and plays a significant role in cefiderocol transport [170]. Notably, catechol‐ and hydroxamate‐based antibiotic conjugates induce the expression of related TonB‐dependent receptors, consistent with siderophores’ physiological roles [171]. This suggests that siderophore–antibiotic drugs may exhibit a sensitivity profile in *P. aeruginosa* populations characterized by an initial increase in susceptibility, but this supposition has not been verified.

As these agents rely on TonB‐dependent iron uptake for cellular entry, the attenuation of activity can arise via structural or regulatory changes in iron‐transport pathways [172]. For cefiderocol, both experimental evolution and clinical reports support a combined trajectory involving iron‐uptake remodeling together with classical β‐lactam resistance determinants, highlighting that “entry” and “payload” mechanisms may synergize in resistance development [173, 174]. For iron‐uptake remodeling, resistance can arise via structural or regulatory changes in TonB‐dependent iron‐uptake pathways. However, one JAC reported that mutations in the upstream regulatory region, which can lead to the downregulation of iron‐uptake transporters and resistance to cefiderocol, are not common in clinical settings, suggesting the possibility of fitness cost [175]. For other siderophore–antibiotic conjugates, direct resistance‐evolution evidence remains limited. However, the documented redundancy in ferri‐enterobactin uptake suggests a higher genetic barrier than vectors relying on a single receptor, while still allowing stepwise attenuation through receptor/pathway remodeling [152, 176].

#### 3.3.3. Broader Trojan Horse Strategies

The “Trojan Horse” strategy extends beyond siderophores to other molecules that induce bacterial uptake. For instance, D‐mannose and D‐glucose grafted onto β‐cyclodextrin, conjugated with erythromycin, rifampicin, or ciprofloxacin, reduced MICs against PAO1. Notably, CD‐MAN‐CIP achieved an MIC of 0.088 mg/L, lower than free ciprofloxacin’s 0.25 mg/L [177]. Additionally, a strategy inducing specific *P. aeruginosa* strains to outcompete wild‐type *P. aeruginosa* has been proposed [178], although it remains in early stages, with challenges in introducing defects into specific strains and effectively eliminating *P. aeruginosa*.

### 3.4. QSI‐Antibiotic Adjuvants

**FIGURE 5:**
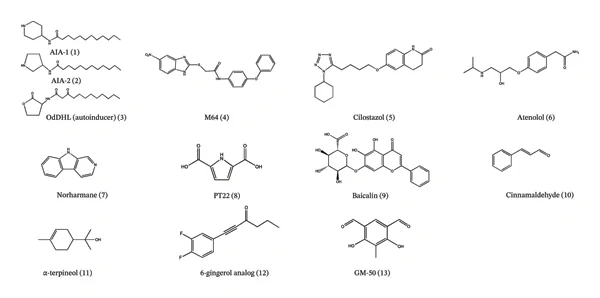
Quorum sensing inhibitors. 1, 2, 4: autoinducer analogs. 3: autoinducer. 5, 6: repurposed drugs. 7, 8: fungal extracts. 9–11: herbal extracts. 12, 13: modified natural QSIs.

QS is a critical mechanism by which *P. aeruginosa* communicates at high cell densities, activating QS genes that modulate pathogenicity and regulate biofilm formation [2]. QS plays a significant role in *P. aeruginosa*’s adaptive resistance, substantially reducing the efficacy of certain antibiotics against bacterial communities [179]. Consequently, inhibiting QS has emerged as an effective strategy to enhance antibiotic susceptibility in *P. aeruginosa*. However, multiple factors—including pharmacokinetic liabilities, toxicity, the intrinsic complexity of QS regulation, and the potential for *P. aeruginosa* to evolve resistance or bypass to QS interference—substantially impede the clinical translation of QSIs; these issues are noted throughout this section and discussed in a consolidated manner at the end. Given the diversity of QSIs, this section focuses on QSIs that can synergize with antibiotics and divides them into six groups by discovering sources. The general mechanism is shown in Figure 1, and all the QSIs are summarized in Table 4 and Figure 5.

**TABLE 4:** Quorum sensing inhibitors.

Compound	Target	Stage	Antibiotic adjuvant effects (reported^∗^)
AIA‐1, AIA‐2	LasR [180]	Preclinical	Azithromycin and clarithromycin [180]
M64	PqsR [181]	Preclinical	Tetracycline [181]
Cilostazol	LasR, RhlR, PqsR [182]	Preclinical	Cefepime [182]
Atenolol	QscR [183]	Preclinical	NA
KS8 fungal supernatant	NA^∗∗^	Preclinical	Tobramycin [184]
Norharmane	PqsA [185]	Preclinical	Polymyxin [185]
PT22	Likely binds LasI, LasR, RhlI, RhlR, and PqsR [186]	Preclinical	Gentamicin [186]
Baicalin and cinnamaldehyde	NA	Preclinical	Tobramycin [187]
α‐Terpineol	Likely LasR receptor [188]	Preclinical	Gentamicin [188]
6‐Gingerol analog	RhlR [189]	Preclinical	Tobramycin [189]
GM‐50	RhlR [190]	Preclinical	Aztreonam [190]

*Note:* Synergy with other antibiotics may exist but has not been reported in the referenced literature.

^∗^Data compiled from published studies.

^∗∗^Have not been reported.

#### 3.4.1. Autoinducer Analogs

Using autoinducer analogs to interfere with *P. aeruginosa*’s QS is a promising strategy. The analogs AIA‐1 and AIA‐2 inhibit QS while increasing the hydrophobicity and permeability of *P. aeruginosa*’s cell surface, enhancing the activity of azithromycin and clarithromycin at 32 μg/mL [180]. High working concentration may lead to their limited follow‐up and translational progression. In 2014, Starkey and colleagues discovered a promising benzamide‐benzimidazole hybrid family of PqsR inhibitors, among which, the leader compound M64 significantly enhances ciprofloxacin’s ability to disrupt *P. aeruginosa* biofilms [191]. In vitro, M64 inhibited PQS and HHQ in the sub‐micromolar range (IC_50_ = 200 and 350 nM, respectively) [192]. In vivo, M64 was also shown to potentiate ciprofloxacin and improve survival in mouse models of *P. aeruginosa* lung infection and burn infection [191]. While no single “fatal flaw” has been explicitly established, the dosing regimen required for efficacy—intravenous administration formulated with 15% cremophor, twice daily for multiple days in mouse models [191]—suggests potential challenges related to solubility and/or half‐life. Thus, although M64 itself appears unlikely to be a straightforward clinical candidate, it represents an important milestone in QSI design. More recent analogs derived from M64, including D88 (solubility 490 μM in PBS) and the indole‐naphthalene compound SPR00305, have been positioned as promising QSIs because they improved survival in lung‐ and burn‐infection mouse models via subcutaneous injection or oral dosing, respectively [193]. Notably, antibiotic‐synergy data for these two QSIs have not yet been reported.

#### 3.4.2. Repurposed Drugs

Repurposing existing drugs for new applications can accelerate the development of clinically viable QSIs, significantly shortening research timelines. Cilostazol [182] and Atenolol [183] were reported to be QSIs that can synergize with antibiotics. However, their effective QSI concentration is high [183], limiting its practical utility as a QSI. But they can provide structural insights for further development.

#### 3.4.3. Fungal Extracts

For unknown reasons, fungi metabolites are more likely to contain QSI. Busetti and colleagues identified KS8, a marine fungus that inhibits *P. aeruginosa*’s QS. Subsequent studies showed that both the supernatant and crude extract of KS8 inhibit QS and synergize with tobramycin, reducing its MIC by tenfold [184]. However, the specific active components and their molecular targets remain unidentified. Similarly, Chen and colleagues, from the Second Institute of Oceanography, SOA, China, screened a library of over 300 deep‐sea bacterial metabolites and identified norharmane, which inhibits PqsA to suppress QS and synergizes with polymyxin [185]. Plant endophytic fungi are another source of QSIs. PT22, an active compound extracted from an endophytic fungus in *Areca catechu* L., reduces multiple QS signaling molecules and synergizes with gentamicin, significantly enhancing its biofilm‐disrupting activity [186]. Because these QSIs generally require relatively high working concentrations [184–186], and each carries a major liability (KS8 is available only as a crude extract; long‐term exposure to norharmane may pose central nervous system toxicity risks [194]; and PT22 appears to fall between additivity and true synergy [186]), they have seen limited classical small‐molecule optimization to date.

#### 3.4.4. Herbal Extracts

Herbal extracts such as baicalin and cinnamaldehyde inhibit QS by interfering with acyl‐homoserine lactone (AHL) signaling, synergizing with tobramycin at 100 and 250 μM, respectively [187]. α‐Terpineol, a plant essential oil, inhibits QS and exhibits potent biofilm‐clearing effects when combined with gentamicin at mg/mL‐range working concentrations [188]. As a class, herbal extract QSIs often exhibit hallmark features such as requiring high concentrations, engaging multiple targets, and showing relatively low affinity properties that may reflect their origin as plant secondary metabolites, which are frequently polypharmacological [195]. Nevertheless, a subset of more promising natural‐product QSIs may warrant deeper mechanistic study and rational improvement, as discussed in the following section [196].

#### 3.4.5. Modified Natural QSIs

Ham and colleagues introduced difluoro and alkynone groups to 6‐gingerol, significantly enhancing its binding affinity to the RhlR receptor and its QS inhibitory activity. Although it inhibited PAO1 biofilm formation at > 3 μM without affecting growth, it can synergize with tobramycin at 1 μM [189]. Given the encouraging activity improvements achieved with these modifications, additional 6‐gingerol analogs have since been developed and reported [197]. Bernabè and colleagues, inspired by coumarin and chromone compounds, synthesized a series of phenolic derivatives and identified GM‐50, which inhibits QS via RhlR and synergizes with aztreonam at 100 μM [190].

#### 3.4.6. Nanoparticle‐Based Delivery Systems

In 2014, solid lipid nanoparticles (SLNs) were found to significantly enhance the penetration of QSIs into artificial sputum, enabling controlled release and increasing the antivirulence activity of a QSI (2‐heptyl‐6‐nitro‐4‐oxo‐1,4‐dihydroquinoline‐3‐carboxamide) by sevenfold [198]. Researchers also explored the codelivery of QSIs and antibiotics using SLNs. Ofloxacin in SLNs reduced the MIC of free ofloxacin by twofold, while ofloxacin–eugenol SLNs reduced it by approximately sixfold [198]. Another group synthesized gentamicin/acylase nanospheres using 0.1% Tween 80 and commercial sunflower oil. Acylase is a QSI enzyme. At a concentration of 1 × 10^13^ NSs/mL, gentamicin nanospheres reduced *P. aeruginosa* counts, whereas at a concentration of 0.25 × 10^13^ NSs/mL, gentamicin/acylase nanospheres completely eliminated *P. aeruginosa* cells [199].

#### 3.4.7. Translational Barriers and Future Prospects for QSIs

QSIs can suppress QS in *P. aeruginosa*, thereby reducing the production of virulence factors and biofilm formation. When used alone, QSIs may mitigate host damage driven by virulence factors and facilitate immune‐mediated clearance; when combined with antibiotics, they may also reduce antibiotic resistance in *P. aeruginosa* [192]. However, the clinical translation of QSIs faces substantial barriers. Firstly, *P. aeruginosa* can evolve escape from QSI‐based interventions, the QSI furanone C‐30 rapidly lost its tobramycin potentiating activity against biofilms during experimental evolution [200]. Secondly, loss‐of‐function LasR variants commonly arise during chronic CF infections and are linked to altered host inflammatory responses, suggesting that LasR‐targeted QSIs may be less effective or even mechanistically mismatched in late stage disease [201]. In parallel, chronic infection can rewire QS hierarchies such that RhlR activity becomes partially independent of LasR [202], implying that the “right” QSI target may differ across infection stages and genotypes, complicating clinical use. Finally, the mechanistic basis of QSI‐antibiotic synergy remains context dependent and cannot be clearly assumed [203]. Systematic profiling indicates that QS signal disruption alters susceptibility across many antibiotics but not uniformly synergistically [204]. Some QSIs even show an ambivalent antivirulence profile against *P. aeruginosa* clinical strains (including persistent infection inducing concerns) [192, 205]. Therefore, the overall therapeutic impact of QSIs—and the mechanistic basis and generalizability of QSI–antibiotic interactions—still requires clearer definition to better support rational clinical development.

In our view, QSIs remain a promising class of antibiotic adjuvants. Potential use cases may include adjunctive treatment at the first episode of P. aeruginosa infection, where transient suppression of bacterial expansion and QS‐driven virulence could enable conventional antibiotics together with host immunity to achieve clearance [206, 207]. In addition, for acute *P. aeruginosa* infections in patients with limited physiological reserve or acute exacerbation in patients with long‐term chronic *P. aeruginosa* infection, short‐term QSI administration might rapidly dampen bacterial growth and virulence‐factor production and augment antibiotic efficacy, with the aim of reducing early mortality [191, 208].

### 3.5. Persister Cell Awakening Strategies

Within the biofilm formed by *P. aeruginosa*, oxygen and nutrients are distributed in a gradient, with basal cells residing in a hypoxic environment, leading to the formation of persister cells. These persister cells exhibit increased antibiotic tolerance due to low cellular activity, altered metabolic pathways, and the production of phenazine compounds [209]. Upon antibiotic withdrawal, persister cells within the biofilm can revert to an active, proliferative state, making *P. aeruginosa* infections challenging to eradicate [49]. Therefore, activating persister cells within biofilms and combining this with antibiotic treatment is a promising strategy for eliminating *P. aeruginosa* infections. Notably, studies have shown that *P. aeruginosa* cells on the biofilm surface are more susceptible to antibiotics targeting bacterial metabolism (e.g., ciprofloxacin) but more resistant to antibiotics that disrupt cell structures (e.g., polymyxin). Conversely, low‐metabolism *P. aeruginosa* cells at the biofilm base exhibit the opposite susceptibility profile [210]. Additionally, research on antibacterial agents has revealed that persister cells are more sensitive to chlorine‐based disinfectants and silver ions compared to active cells but are less responsive to tobramycin [211]. Current antibiotic adjuvants targeting persister cells focus on three main strategies: (1) increasing oxygen concentration within the biofilm, (2) improving nutrient supply within the biofilm, and (3) activating persister cells with signaling molecules. The general mechanism is shown in Figure 1, and all the strategies are summarized in Table 5.

**TABLE 5:** Persister cell awakening strategies.

Persister activation method	Activation principle	Stage	Effect
Weak direct current	Improves hypoxia [212, 213]	Preclinical	Synergizes with industrial biocides [213] or tobramycin [212]
Hyperbaric oxygen environment	Improves hypoxia [214]	Preclinical	Reduces tobramycin’s bactericidal concentration against biofilms by over 50% [214]
MCPGaGP	Improves hypoxia [215]	Preclinical	Significantly alleviates biofilm hypoxia, activates and kills persister cells [215]
Coadministered mannitol	Improves nutrient supply [216, 217]	Preclinical	Activates persister cells, synergizes with tobramycin [216], ciprofloxacin [217]
cis‐DA	Signaling molecule [218]	Preclinical	Induces biofilm dispersion, activates persister cells, synergizes with ciprofloxacin, tobramycin, and tetracycline [218]

*Note:* MCPGaGP: mesoporous silica, catalase, Ga^3+^. cis‐DA: cis‐2‐decenoic acid.

#### 3.5.1. Increasing Oxygen Concentration Within the Biofilm

As early as 1999, Stewart et al. suggested that weak direct current enhances antibiotic efficacy against *P. aeruginosa* by generating oxygen through electrolysis, thereby increasing oxygen levels within the biofilm [212]. In 2019, another study found that hyperbaric oxygen environments increase the oxygen content in *P. aeruginosa* biofilms. While hyperbaric oxygen alone promotes *P. aeruginosa* growth, its combination with TOB reduces the bactericidal concentration required to target biofilms by over 50% [214]. In a 2024 study, He and colleagues developed an innovative material to activate persister cells. They embedded catalase in mesoporous silica, protected the catalyst with polydopamine and cross‐linked Ga^3+^ ions and maltohexaose on the surface to create MCPGaGP. In the presence of H_2_O_2_, MCPGaGP releases catalase and Ga^3+^. Catalase catalyzes the production of O_2_, alleviating hypoxia within the biofilm, activating persister cells, and enhancing their uptake of Ga^3+^ [215].

#### 3.5.2. Improving Nutrient Supply Within the Biofilm

Certain sugars have been used to activate persister cells. Mannitol, already employed for mucus clearance in cystic fibrosis patients, was further investigated by Barraud et al., who found that it increases persister cell metabolism, reversing their phenotype and enhancing TOB’s bactericidal activity against them [216, 217].

#### 3.5.3. Signaling Molecules to Activate Persister Cells

The signaling molecule cis‐2‐decenoic acid (cis‐DA) induces biofilm dispersion while awakening persister cells, upregulating proteins related to metabolism, protein repair, transport, and ATP synthesis. Consequently, cis‐DA enhances the bactericidal activity of ciprofloxacin, TOB, and tetracycline against persister cells [218].

#### 3.5.4. Other Therapies Targeting Persister Cells

Despite the significant role of persister cells in persistent *P. aeruginosa* infections, research on targeted therapies remains limited. Beyond improving hypoxia, enhancing nutrient supply, and using signaling molecules to activate persister cells, other strategies include sequential treatment with aztreonam and TOB [219], as well as the combined use of polymyxin (targeting persister cells) and ciprofloxacin (targeting metabolically active *P. aeruginosa*) [210].

#### 3.5.5. Translational Challenges for Persister Cell Awakening Strategies

Unlike traditional adjuvants such as antibiotics, membrane permeabilizers, or EPIs—where in vitro susceptibility testing can sometimes provide partial guidance for in vivo use—persister cell awakening strategies primarily aim to modulate bacterial phenotypic state and adaptive tolerance. As a result, they often lack standardized clinical indications and validated diagnostic pathways; if translated into practice, their use could easily drift toward empiricism (e.g., based on infection duration, clinical phenotype, and a limited set of advanced assays such as genotyping). More importantly, these strategies are highly timing‐dependent and can carry “reverse‐risk” scenarios: Increasing oxygenation may enhance antibiotic killing within biofilms, yet hyperbaric oxygen itself can promote *P. aeruginosa* growth [214]; cis‐DA can induce biofilm dispersion and awaken persisters but may concurrently increase the risk of dissemination [218]. These considerations suggest that, unless tightly coupled to adequate antibiotic exposure, interventions may preferentially “awaken/accelerate growth” rather than achieve eradication. Even if persister awakening strategies can help clear chronic *P. aeruginosa* infections, clinical adoption will require careful balancing of benefit against treatment‐associated risks and the ongoing vulnerability of predisposed patients to reinfection.

## 4. Conclusions

The treatment of *P. aeruginosa* infections remains a formidable challenge in clinical medicine. This bacterium exhibits multiple resistance mechanisms, such as low outer‐membrane permeability, efflux pump overexpression [13], QS activation, and persister cells [12]. These complex resistance mechanisms significantly diminish the efficacy of conventional antibiotics, particularly in chronic infections like those in cystic fibrosis patients, where biofilms and persister cells contribute to the persistence and recalcitrance of infections [47].

Antibiotic adjuvants offer a promising strategy to enhance antibiotic efficacy and mitigate the development of resistance. These adjuvants counteract bacterial resistance through mechanisms such as increasing outer‐membrane permeability [47], suppressing efflux pumps [2], employing the “Trojan Horse” strategy via siderophores [161], inhibiting QS [180], and activating persister cells [215]. The concept of traditional antibiotic adjuvants is rather narrow, and recent advancements require a broader definition. An antibiotic adjuvant is a compound coadministered or covalently linked with antibiotics to enhance the activity of the antibiotic [56]. The core principle of antibiotic adjuvants is to synergize with specific antibiotics or antibacterial agents through targeted mechanisms [220]. Their objectives include overcoming resistance [220], enhancing antibiotic potency [53], or targeting specific bacterial phenotypes [215]. In vitro and animal model studies have demonstrated significant antibacterial potential, providing a theoretical foundation for developing novel antibacterial drugs. However, clinical applications face challenges, mainly high effective concentration, toxicity, complex mechanisms, additional clinical indication requirements, and continued reliance on combination antibiotic therapies as the optional short‐term strategy. Moreover, uncertain returns and high development costs make antibiotic adjuvant programs particularly prone to funding gaps and limited industrial investment, increasing reliance on public sector and academic efforts. Nevertheless, as *P. aeruginosa* resistance escalates, the development of antibiotic adjuvants will be increasingly prioritized.

Developing new antibacterial agents and alternative therapeutic strategies is a long and challenging journey. Future research should focus on optimizing existing strategies while exploring novel approaches, improving current antibiotics, and developing adjuvants to keep pace with the rapid evolution of *P. aeruginosa* resistance. These efforts hold promise for achieving breakthroughs in treating *P. aeruginosa* infections, improving patient outcomes, and alleviating the public health burden.

## Funding

This work was supported by the R&D Program of Guangzhou National Laboratory (No. GZNL2024A02003), the Shanghai Municipal Science and Technology Major Project (No. ZD2021CY001), the National Key Research and Development Program of China (No. 2024YFC3044400), and the Construction of Multi‐Disciplinary Treatment System for Severe Pneumonia (No. W2020‐013).

## Conflicts of Interest

The authors declare no conflicts of interest.

## Data Availability

Data sharing is not applicable to this article as no datasets were generated or analyzed during the current study.
